# Assessment of attenuation properties for SLA and SLS 3D-printing materials in X-ray imaging and nuclear medicine

**DOI:** 10.1016/j.zemedi.2024.02.003

**Published:** 2024-03-12

**Authors:** Stefan Weber, Andreas Block, Felix Bärenfänger

**Affiliations:** a TU Dortmund, August-Schmidt-Straße 1, Dortmund 44227, Germany; b Klinikum Dortmund, Beurhausstraße 40, Dortmund 44137, Germany

**Keywords:** 3D-Printing, Nuclear medicine, X-ray, Mass-attenuation, Correction factors, Formlabs

## Abstract

In recent years, access to 3D printers has become increasingly affordable. Alongside industrial and private applications, the significance of 3D printing in the clinical context is also growing. For instance, 3D printing processes enable the production of individual anatomical models that can be used to support patient communication or aid in surgical planning. While filament 3D printing is common, stereolithography (SLA) and selective laser sintering (SLS) printing processes offer higher precision. For the use of 3D printing materials in radiology, understanding their attenuation properties concerning ionizing radiation is crucial. Polymethyl methacrylate (PMMA) serves as an important reference material for radiological applications in this regard.

In this research, linear- and mass attenuation coefficients of 38 SLA-/SLS-materials from Formlabs (Somerville, Massachusetts, USA) and PMMA will be determined through intensity measurements in nuclear medicine for the radionuclides technetium-99 m and iodine-131, as well as for X-ray imaging in the range of 60 kVp - 110 kVp tube voltage. Based on the mass attenuation coefficients, correction factors in respect to PMMA will be calculated for each material. A significant number of materials exhibit a deviance within approximately ±5% in respect to PMMA regardless of radiation energy. However, certain materials from the dental and industrial application show deviances up to +500% at the lower end of radiation energy spectrum. In conclusion, most materials can be considered equivalent to PMMA with only minor adjustments required. Materials with high deviances can be utilized as high-contrast materials in custom X-ray phantoms.

## Introduction

3D printing technology has made significant progress in recent years and has become a crucial tool in the medical field. In addition to filament printing, SLA- and SLS-printing are particularly noteworthy 3D printing processes. In SLA printing, liquid photopolymers (resins) are cured under UV light in layers well below 100 μm thickness. Meanwhile, SLS printing is based on the partial melting and subsequent curing of fine powders (SLS powder) of varying composition (e.g., plastic, ceramic, glass, metal) with a layer thickness similar to that of SLA. These processes are increasingly utilized in medical research and practice due to their fine resolution. For example, 3D printing processes in prosthetics and orthopedics enable the production of customized implants [1] and prostheses [2]. In dentistry, the technology is used to manufacture crowns, bridges, orthodontic braces, and surgical templates [3]. However, the application of 3D printing in medical physics is of particular interest in the fields of X-ray imaging, nuclear medicine, and radiation-/brachytherapy. It is used for fabricating custom phantoms [4] and applicators [5] for therapy planning and application. In nuclear medicine, 3D printing can be used to determine organ-specific SPECT calibration factors [6] and for phantom-based optimisation of image quantification for SPECT/CT [7].

The fine resolution provided by SLA and SLS makes these processes valuable in medical research and practice. For the application in quality assurance for medical imaging or even for treatment planning in radiation therapy, a comprehensive knowledge of the attenuation properties of the materials is crucial. Radiological attenuation properties depend on factors such as the material’s thickness, density, chemical composition, and the energy of the radiation used. Using materials with unknown radiological attenuation properties can lead to a large bias in dose calculations.

The radiological attenuation properties of materials strongly correlate with their density. Therefore, to ensure better comparability between different materials, they are often expressed as mass attenuation coefficients. By comparing them with those of a well-characterized reference substance, it becomes possible to classify the attenuation properties. Polymethyl methacrylate (PMMA), also known as acrylic glass, has become a widely accepted reference substance due to its fixed chemical composition, consistent density, transparency, and cost-effectiveness. Alongside water, another important reference substance, PMMA is advantageous as a solid material that is relatively easy to process. Its well-known radiological attenuation properties make it an important reference material for various imaging techniques.

A variety of vendors for SLA 3D printers (such as Formlabs, Anycubic, Creality, Elegoo, etc.) are available, and many of them have their own resin product ranges. Formlabs, in particular, offers the most extensive range with over 35 different materials [8], suitable for various applications ranging from jewelry to engineering, as well as resins specifically designed for medical and dental purposes. While there has been extensive research in the characterisation of fused-deposit-modelling (FDM) materials [9], [10] in the past, there is currently no study that has investigated such a wide range of SLA and SLS materials as has been done in this research.

The main objective of this research was to investigate the attenuation properties of a wide selection of Formlabs SLA and SLS materials. To avoid redundancy, not all materials and color variants offered by Formlabs were examined. A list of the investigated materials can be found in Table 2. The research focused on comparing these properties with those of PMMA. Establishing a connection between the attenuation properties of these 3D printing materials and PMMA opens up a wide array of applications. By determining the alignment of these materials’ attenuation properties with PMMA, the study aimed to identify viable substitutes and alternatives for PMMA in medical applications. The investigation encompassed specific X-ray imaging scenarios, spanning tube voltages from 60 kVp to 110 kVp, as well as the two most widely used radionuclides in nuclear medicine: technetium-99 m (Tc-99 m, 140.5 keV) [11] and iodine-131 (I-131, 364.5 keV) [12]. As a result, this research provides a series of correction factors for mass attenuation coefficients regarding PMMA. Both mass attenuation coefficients and linear attenuation coefficients can be found in the Appendix.

## Material and methods

### Production of sample objects

38 resin and SLS materials were investigated for their attenuation properties. These materials were selected from the Formlabs product range, including BioMed, dental, SLS powder, technical, and standard resin series. Test objects for the ClearV4 and Elastic50A materials were printed on-site at Klinikum Dortmund using a Fromlabs Form 3 printer, while all other test objects were generously provided by Formlabs. Test objects made from PMMA were manufactured from PMMA plates by the technical workshop of TU Dortmund.

For each material, three cylindrical test objects were manufactured with a nominal diameter of 90 mm and nominal thicknesses of 10 mm, 20 mm, and 40 mm, respectively. To determine the precise object thicknesses and mass densities, the geometric properties (thickness and diameter) of all test objects were measured using a caliper gauge (0.05 mm scale interval). The weight of each test object was measured using a precision balance (apotec Quintix recipe scale; 10 mg scale interval). Assuming laboratory conditions (20°C, 1 atm), the measured weights were corrected for air buoyancy to obtain the mass. The material-specific mass densities $\rho_{m}$ were determined from the mean values of the mass densities of the individual objects of a material and are listed in Table 2.

### Experimental setup in X-ray imaging

The measurements in X-ray imaging were conducted using a Siremobil Compact L c-arm machine (Fa. Siemens, Erlangen, Germany). The inherent filtration of the X-ray tube is specified with a fixed ≤3 mm Al equivalent. The tube voltage can be regulated within a range of 50–110 kVp, with the anode current automatically following and not adjustable separately. The concentric primary collimation provides a field size of approximately 4 cm in diameter in the image receptor plane at maximum collimation. An overview of the measurement program is presented in Table 1.

**Table 1:** Radiation qualities used in X-ray imaging. Al-HVL equivalents for 60 kVp, 80 kVp and 99 kVp are intrapolated.

Tube voltage [kVp]	60	70	80	90	99	110
Anode current [mA]	0.7	1.1	1.5	2.0	2.5	3.0
Al-HVL [mm]	3.58	4.19	4.78	5.03	5.92	6.80

**Fig. 1:**
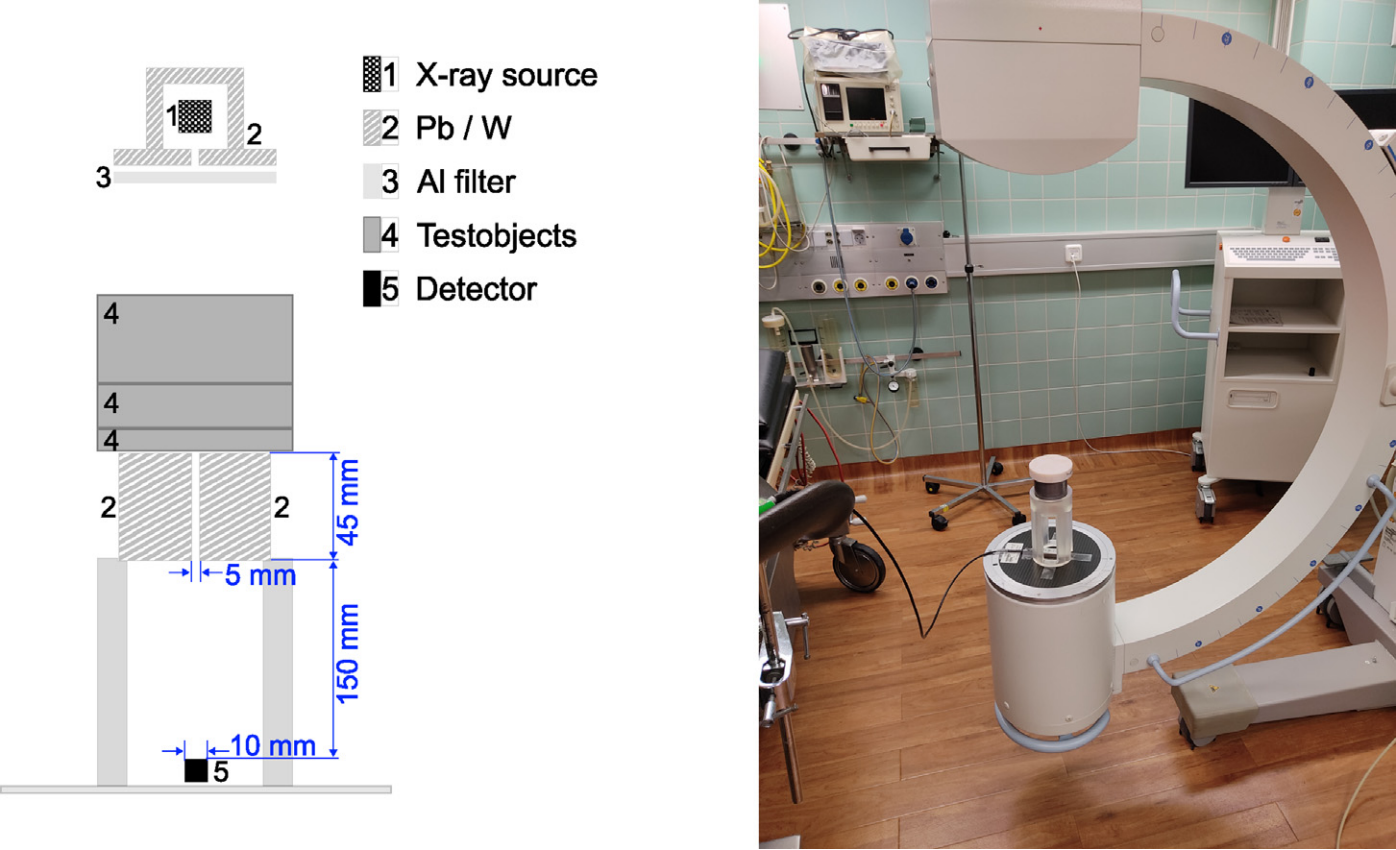
Left: Sketch of the experimental setup in X-ray imaging. Right: Experimental setup with PMMA test object.

The detector and experimental setup were centrally placed on the image receiver (see Fig. 1). The base of the experimental setup was 3D-printed using ClearV4 resin. To further reduce the field size and cover only the detector area, additional collimation to a field size of approximately 5 mm in diameter was implemented using a Pb/W-collimator of 45 mm thickness. The intensity measurements were recorded as dose rate $\overset{˙}{D}$ using an ”XR - Multidetector” (Si-PIP photoelectrode on a Si-based detector) from IBA Dosimetry GmbH (Schwarzenbruck, Germany), which has a detector area with a diameter of 1 cm. To ensure correct positioning and alignment of the experimental setup, the collimator’s position within the setup was adjusted to maximize the measured dose rate at a tube voltage of 90 kVp. This tube voltage provided the best contrast for the interior of the detector, allowing the detector chip to be visually distinguished within the detector and providing orientation for optimal alignment of the experimental setup.

Since the anode current is linked to the tube voltage in the utilized system, adjustments to improve the signal-to-noise ratio (SNR) at 50 kVp were not possible, leading to the exclusion of 50 kVp measurements. Additionally, measurement series with dose rates systematically ⩽1 μGy/s were not considered due to SNR limitations. Consequently, for some materials, a complete measurement series with eight thicknesses (0–7 cm) could not be obtained for smaller tube voltages, resulting in fewer values included in the fit.

### Experimental setup in nuclear medicine

The measurement program in nuclear medicine encompasses two of the most commonly used radionuclides in imaging (Tc-99 m, 140.5 keV) [11] and radionuclide therapy (I-131, 364.5 keV) [12]. Tc-99 m was utilized as a radiation source in a buffer solution, while I-131 was used either in capsule form or in a buffer solution, depending on availability. Test measurements with different volumes of buffer solutions (2 ml, 10 ml and 20 ml) showed no measurable effect of the source volume on the results for this particular setup. However, to minimize any potential influence on the measurement results due to volume effects in the liquid sources, consistent volumes of approximately 2 ml of buffer solution were used for all measurements. The same type of vile was used for both liquid and capsule sources. Liquid and capsule sources were not differentiated. The intensities were recorded as count rates (counts/min - cpm) using a NaI(Tl)-scintillation detector with integration across the photopeak (multiple of 2.5 times the full-width at half-maximum [FWHM]). These translate into energy windows of 140±16 keV for Tc-99 m and 364±41 keV for I-131.

**Fig. 2:**
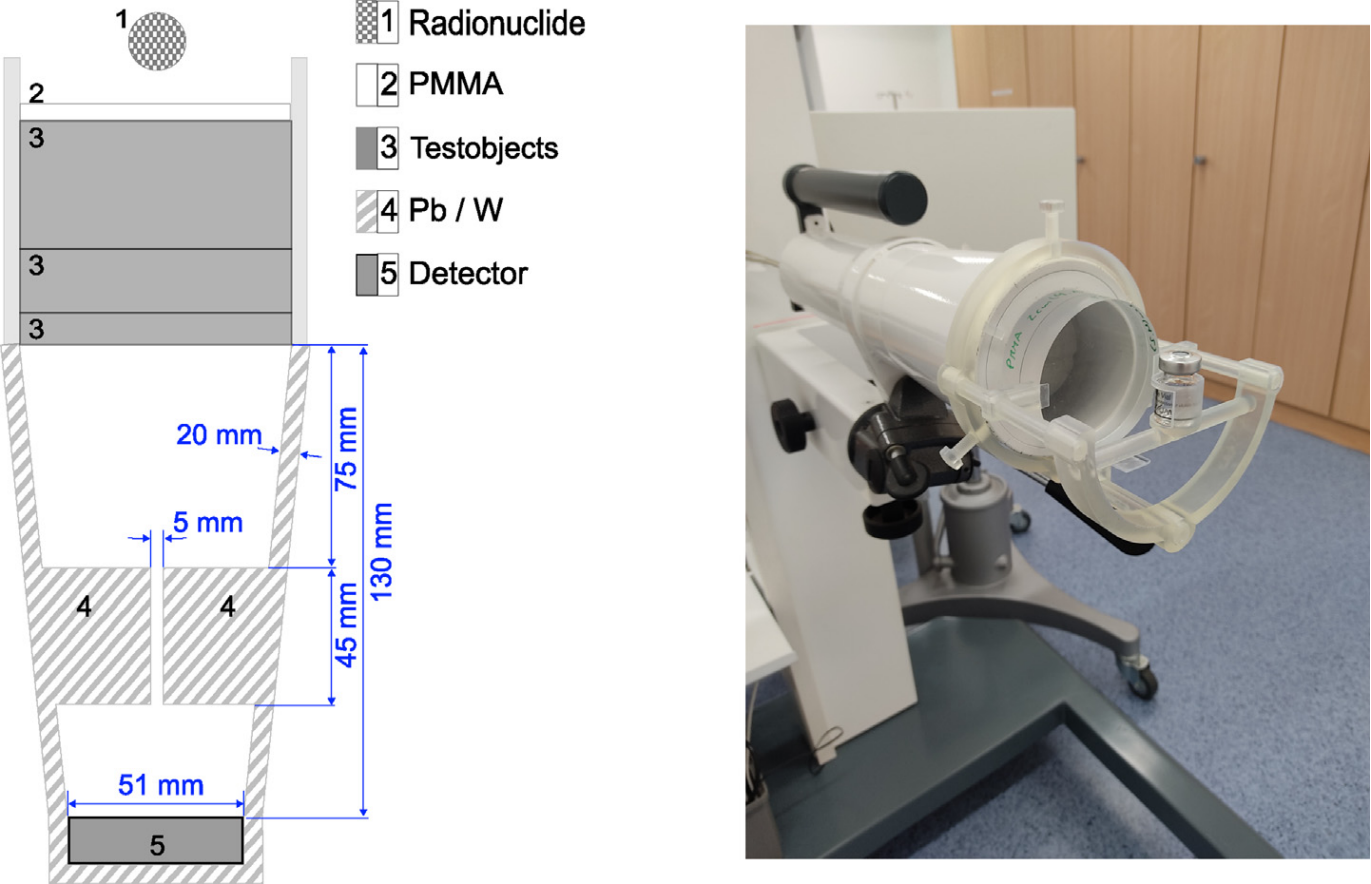
Left: Sketch of the experimental setup in nuclear medicine. Right: Experimental setup with PMMA test object.

The experimental setup was 3D-printed using ClearV4 Resin and designed to be directly attached to the shielding of the measurement station. The test objects were sized to completely cover the opening of the shielding. A collimator (thickness 45 mm, opening 5 mm) from Pb/W was inserted into the shielding. The setup is shown in Fig. 2. Each individual measurement was corrected for detector dead time and radioactive decay according to the law of decay, with reference to the start of the measurement series. For measurements with I-131, in addition to the investigated test objects, an electron filter (5 mm PMMA) was inserted between test objects and radiation source. For PMMA itself, the zero thickness was replaced by the 5 mm electron filter and included in the fit. For all other materials, the additional radiation attenuation caused by the electron filter was corrected using the measured linear attenuation coefficient of PMMA. To correct for background radiation, a series of measurements was performed for each material just before the main measurements.

### Calculation of the mass attenuation coefficients and correction factors

To determine the linear attenuation coefficients $\mu_{L}$, the radiation intensities were measured for each material as a function of the irradiated thicknesses, aiming to obtain a full set of data points covering a range from 0 to 70 mm for each material. However, in some cases, measurements in X-ray imaging had to be limited, depending on the attenuation properties and tube voltage. The measured intensities were corrected for background radiation and, in the case of measurements with radionuclides, for decay. An exponential fit was then performed using the attenuation law ( Eq. 1) to determine the linear attenuation coefficients. The relative uncertainties σ of both *x*-inputs (material thickness) and *y*-inputs (intensities) were included in the fit as weights with $1/|\sigma_{x/y}{|}^{2}$. For measurements in X-ray imaging, sufficient data points for a fit could not be obtained for Permanent Crown (60 kVp, 70 kVp) and Temporary CB (60 kVp, 70 kVp, 80 kVp) due to their high attenuation properties. In these cases, linear attenuation coefficients were calculated directly via Eq. 1 using intensities from 0 cm and 1 cm object thickness.

(1) $$I(x)=I_{0}\exp(-\mu_{L}\cdot x)$$

From the determined linear attenuation coefficients and mass densities, the mass attenuation coefficients $\mu_{\rho }$ were directly calculated using Eq. 2. Subsequently, these calculated mass attenuation coefficients were used to determine the correction factors $k_{i}$ (Eq. 3) relative to PMMA.

(2) $$\mu_{\rho }=\mu_{L}/\rho$$

(3) $$k_{i}=\mu_{\rho_{\mathrm{PMMA}}}/\mu_{\rho_{i}}$$

The correction factors $k_{i}$ are closely linked to the relative deviation $f_{i}$ via Eq. 4

(4) $$f_{i}=1-k_{i}$$

Data analysis was conducted using OriginPro 2022b. The error analysis in this study adheres to the guidelines outlined in JCGM 100:2008 (GUM) [13].

## Results

An overview of the investigated materials as well as the determined mass densities can be found in Table 2. The correction factors are summarized in Table 5 (nuclear medicine) and Table 6 (X-ray). Mass attenuation coefficients and linear attenuation coefficients are available in the appendix.

**Table 2:** List of all examined materials and their area of application. Uncertainties given in the parenthesis refer to the last digits.

Material	Series	$\rho_{m}$ [g/cm^3^]	Material	Series	$\rho_{m}$ [g/cm^3^]
PMMA	reference material	1.183(3)	Nylon11	SLS powder	1.006(3)
Black	standard resin	1.166(5)	Nylon11 CF	SLS powder	1.049(4)
ClearV4	standard resin	1.170(8)	Nylon12	SLS powder	0.975(7)
Draft	standard resin	1.181(5)	Nylon12 GF	SLS powder	1.259(8)
Grey	standard resin	1.168(4)	TPU90A	SLS powder	1.128(5)
White	standard resin	1.165(4)	BioMed Amber	BioMed	1.186(4)
Castable	technical resin	1.108(5)	BioMed Black	BioMed	1.187(4)
Durable	technical resin	1.126(3)	BioMed Clear	BioMed	1.169(4)
Elastic50A	technical resin	1.08(2)	BioMed Elastic50A	BioMed	1.08(1)
ESD	technical resin	1.131(3)	BioMed Flexible80A	BioMed	1.098(4)
Flexible80A	technical resin	1.108(5)	BioMed White hd	BioMed	1.232(6)
Grey Pro	technical resin	1.165(4)	BioMed White ld	BioMed	1.174(6)
High Temp	technical resin	1.197(4)	Castable Wax	dental	1.188(6)
PU 650	technical resin	1.146(3)	Custom Tray	dental	1.186(3)
PU 1000	technical resin	1.144(4)	Denture Base	dental	1.187(3)
Rigid10k	technical resin	1.697(4)	Denture Teeth	dental	1.181(3)
Rigid4000	technical resin	1.332(3)	IBT	dental	1.128(5)
Tough1500	technical resin	1.147(4)	Model V3	dental	1.190(3)
Tough2000	technical resin	1.187(3)	Permanent Crown	dental	1.475(6)
			Temporary CB	dental	1.490(4)

The test objects for BioMed White displayed two distinctly different densities of 1.23 g/cm^3^ for the 10 mm test object and 1.17 g/cm^3^ for the 20 mm and 40 mm test objects. A second, newly ordered 10 mm test object showed the same density of 1.23 g/cm^3^ as the previous one. This magnitude of variation in density was not observed in other materials. The fact that Formlabs did not provide any insight into the manufacturing process of the test objects provided led to the decision to label the BioMed White test objects as two different materials with the ”low-density” (ld) and ”high-density” (hd) additions. The ”ld” variant aligns more closely with comparable materials such as BioMed Black, BioMed Amber, and BioMed Clear in terms of both density and attenuation properties. Due to the presence of small air cavities at the edge of the 10 mm test object for Custom Tray, its density is slightly reduced. Therefore, it is excluded from the purpose of determining the materials’ mass-density $\rho_{m}$.

In nuclear medicine the determined mass attenuation coefficients of PMMA were compared to literature values [14] for validation of the experimental setup (Table 3). The experimentally determined values agree well with the literature, with deviations smaller than 4% for Tc-99 m and smaller than 7% for I-131.

**Table 3:** Relative deviations of the determined mass attenuation coefficients of PMMA from the literature value in nuclear medicine [14]. Uncertainties given in the parenthesis refer to the last digits.

	Energy	Exp.-value [${\mathrm{cm}}^{2}/g$]	Literature [${\mathrm{cm}}^{2}/g$]	Deviation [%]
Tc-99 m	140.5 keV	0.1441(6)	0.1491	-3.35(40)
I-131	364.5 keV	0.100(2)	0.1074	-6.8(14)

For validation in X-ray imaging, linear attenuation coefficients for PMMA were referenced against data from a comparable study [15] (Table 4). Their measurements at 70 kVp tube voltage, with an inherent filtration of 2.58 mm Al, exhibit a comparable radiation quality to the one used here. The deviation from the reference is less than 5%.

**Table 4:** Relative deviations of the determined linear attenuation coefficient of PMMA at 70 kVp compared to the results of a study with comparable radiation quality [15]. Uncertainties given in the parenthesis refer to the last digits.

Tube voltage	Exp.-value [1/cm]	Reference [1/cm]	Deviation [%]
70 kVp	0.2872(11)	0.30	-4.27(37)

Analysis of the correction factors (Table 5 and Table 6) reveals that most investigated materials closely align with PMMA within a range of ± 5%. In many cases, the deviation is even less than ± 2%. Therefore, these materials can serve as suitable substitutes for PMMA with only minor adjustments to measurements or thickness required. Nevertheless, certain materials exhibit substantial differences in mass attenuation properties compared to PMMA. For instance, the dental resins Permanent Crown and Temporary CB demonstrate deviations of up to  + 500% in X-ray imaging at 60 kVp, rendering them radiopaque. However, as tube voltage and γ-energy increase, their properties approach those of other materials, and excessive deviation can no longer be observed for I-131.

**Table 5:** Correction factors k with respect to PMMA in nuclear medicine for Tc-99 m (140.5 keV) and I-131 (364.5 keV). Uncertainties given in the parenthesis refer to the last digits. Bold: ⩾5% deviation from PMMA Non bold: <5% deviation from PMMA.

	Tc-99 m	I-131
Material	140.5 keV	364.5 keV
BioMed Amber	0.994(13)	1.005(20)
BioMed Black	0.986(13)	0.980(18)
BioMed Clear	1.000(10)	0.985(19)
BioMed Elastic50A	1.016(13)	1.035(42)
BioMed Flexible80A	1.029(17)	0.967(48)
BioMed White hd	1.021(10)	1.020(52)
BioMed White ld	0.999(18)	0.929(34)
Black	1.005(8)	1.018(18)
Castable	0.995(12)	1.019(22)
Castable Wax	1.004(10)	1.030(25)
ClearV4	0.990(10)	0.997(17)
Custom Tray	1.002(12)	0.988(21)
Denture Base	1.017(8)	1.008(17)
Denture Teeth	1.015(9)	1.018(20)
Draft	1.001(9)	0.997(21)
Durable	0.999(10)	1.014(23)
Elastic50A	0.998(15)	0.991(21)
ESD	0.999(9)	0.973(28)
Flexible80A	0.988(13)	1.021(22)
Grey	1.008(12)	1.041(25)
Grey Pro	0.992(10)	0.986(17)
HighTemp	1.052(19)	0.994(18)
IBT	1.002(9)	0.992(28)
ModelV3	1.002(17)	1.036(20)
Nylon11	0.978(9)	1.005(32)
Nylon11CF	1.009(9)	1.005(20)
Nylon12	0.968(13)	0.948(19)
Nylon12GF	1.003(12)	1.042(27)
Permanent Crown	0.698(7)	1.001(20)
PU650	1.001(12)	1.098(40)
PU1000	0.984(9)	0.944(28)
Rigid10k	1.045(13)	1.032(19)
Rigid4000	1.016(9)	1.027(19)
Temporary CB	0.675(6)	1.012(24)
Tough1500	1.015(9)	0.981(29)
Tough2000	1.018(11)	1.001(24)
TPU90A	0.987(10)	0.949(36)
White	1.004(12)	0.965(34)

**Table 6:** Correction factors k of the mass attenuation coefficients with respect to PMMA in X-ray imaging (tube voltages 60-110 kVp). Uncertainties given in the parenthesis refer to the last digits. ☆ Values determined from direct comparative measurements. Bold: ⩾5% deviation from PMMA Non bold: <5% deviation from PMMA

	Tube Voltage					
Material	60 kVp	70 kVp	80 kVp	90 kVp	99 kVp	110 kVp
BioMed Amber	0.978(7)	0.988(7)	0.981(7)	0.989(8)	1.004(12)	0.992(10)
BioMed Black	0.960(9)	0.965(8)	0.959(8)	0.963(8)	0.965(16)	0.964(11)
BioMed Clear	1.012(7)	1.010(7)	1.000(9)	1.006(8)	1.017(12)	1.011(11)
BioMed Elastic50A	1.026(13)	1.022(12)	1.010(13)	1.011(13)	1.012(19)	1.006(14)
BioMed Flexible80A	1.009(9)	1.014(14)	0.995(8)	1.001(8)	1.001(16)	0.995(12)
BioMed White hd	0.863(10)	0.872(12)	0.879(12)	0.886(12)	0.872(29)	0.902(22)
BioMed White ld	0.983(7)	0.992(8)	0.986(10)	0.992(10)	1.001(15)	0.992(12)
Black	0.977(8)	0.981(7)	0.974(8)	0.980(8)	0.991(13)	0.990(11)
Castable	0.997(7)	0.991(7)	0.982(8)	0.986(9)	0.999(13)	0.997(11)
Castable Wax	0.994(7)	0.985(8)	0.979(8)	0.986(8)	1.000(13)	0.999(11)
Clear	0.992(8)	0.996(9)	0.986(9)	1.004(9)	0.994(16)	0.992(12)
Custom Tray	0.992(9)	0.984(9)	0.972(10)	0.982(10)	0.997(14)	0.999(13)
Denture Base	1.008(5)	0.998(6)	0.990(7)	0.996(7)	1.009(11)	1.005(10)
Denture Teeth	0.962(6)	0.969(7)	0.968(7)	0.973(8)	0.987(12)	0.989(11)
Draft	1.010(6)	1.006(7)	0.994(8)	1.000(8)	1.011(12)	0.975(15)
Durable	0.991(6)	0.993(6)	0.986(7)	0.992(7)	1.002(12)	0.999(11)
Elastic50A	0.995(17)	0.999(14)	0.991(14)	0.999(14)	1.007(17)	1.005(15)
ESD	0.996(6)	0.998(6)	0.988(7)	0.996(8)	1.009(12)	1.004(10)
Flexible80A	0.983(8)	0.983(7)	0.979(8)	0.985(9)	0.996(12)	0.993(11)
Grey	0.960(6)	0.964(7)	0.964(7)	0.979(7)	0.983(12)	0.982(11)
Grey Pro	0.952(8)	0.960(7)	0.958(8)	0.967(8)	0.978(12)	0.975(10)
High Temp	1.008(6)	1.008(7)	1.000(7)	1.006(8)	1.016(12)	1.012(10)
IBT	0.996(7)	0.999(8)	0.992(8)	0.995(8)	1.007(12)	1.004(10)
ModelV3	1.014(7)	1.010(8)	0.998(8)	1.000(8)	0.999(13)	0.996(11)
Nylon11	1.033(7)	1.022(7)	1.003(7)	1.004(9)	0.995(13)	0.992(10)
Nylon11CF	1.047(7)	1.034(7)	1.020(8)	1.023(8)	1.031(12)	1.027(10)
Nylon12	1.031(9)	1.019(9)	1.001(10)	0.999(10)	0.993(15)	0.992(11)
Nylon12GF	0.637(12)	0.667(12)	0.690(13)	0.718(14)	0.746(18)	0.755(17)
Permanent Crown	0.190(3)☆	0.187(2)☆	0.189(5)	0.195(7)	0.203(8)	0.211(9)
PU650	1.011(7)	1.015(6)	1.001(7)	1.001(7)	1.002(15)	0.999(10)
PU1000	1.017(7)	1.010(8)	1.000(8)	1.001(8)	1.000(16)	0.997(11)
Rigit10k	0.566(19)	0.612(20)	0.639(20)	0.670(20)	0.692(24)	0.714(22)
Rigit4000	0.751(11)	0.785(12)	0.800(11)	0.828(13)	0.846(17)	0.856(16)
Temporary CB	0.173(3)☆	0.173(2)☆	0.176(2)☆	0.185(7)	0.195(8)	0.202(11)
Tough1500	1.011(8)	1.005(9)	0.994(8)	0.997(8)	0.995(14)	0.993(11)
Tough2000	0.901(9)	0.912(9)	0.908(10)	0.919(9)	0.924(14)	0.927(12)
TPU90A	0.999(8)	0.997(8)	0.984(8)	0.988(8)	0.986(14)	0.986(11)
White	0.974(8)	0.977(8)	0.966(8)	0.971(8)	0.972(13)	0.971(11)

Another group of materials, including Nylon12 GF, Rigid4000, and Rigid10k, deviates significantly from PMMA (up to  + 77% at 60 kVp). Interestingly, these materials are advertised to have a glass component according to the Formlabs material library [8].

Throughout the investigation, none of the examined materials exhibited mass-attenuation properties significantly lower than those of PMMA.

## Discussion

The validation of the experimental setups has identified a systematic error ranging from approximately −3.5% to −7%, depending on the specific setup and radiation source. This error is likely attributed to insufficient collimation. The observed systematic errors affect both linear and mass attenuation coefficients but do not impact correction factors, as they uniformly influence all measurements, canceling out during the calculation of correction factors according to Eq. 3. It is also important to note that the literature values provided by NIST [14], used for the validation in nuclear medicine, are based on simulations using pencil-beam radiation sources, which do not match the radiation source used here. As for the validation in X-ray imaging, while the radiation quality in [15] is comparable, they do not match exactly and the exact differences in radiation qualities and PMMA mass densities are unknown. It is also important to emphasise the difference between the polyenergetic narrow beam geometry for the attenuation coefficients in X-ray imaging and the monoenergetic narrow beam geometry for the attenuation coefficients in nuclear medicine.

Across the investigated energy spectrum, most materials can be considered as PMMA equivalents with minor corrections, however, BioMed Clear and ClearV4 are particularly good candidates as both materials are very close to PMMA not only in terms of attenuation but also in terms of feel and appearance. Notable exceptions include the dental materials Temporary CB and Permanent Crown, which exhibit high deviations from PMMA in X-ray imaging, rendering them radiopaque at lower tube voltages. Despite these deviations, these materials still hold promise as excellent candidates for the production of custom contrast radiophantoms. The moderate deviation at the lower end of the investigated energy spectrum for glass-compound materials aligns well with literature values of PMMA and Glass (Borosilicate, ”Pyrex”) [14]. It is noteworthy that Rigid10k, despite having the highest density of all the materials investigated, has lower attenuation than Temporary CB and Permanent Crown in the X-ray application and for Tc-99 m. This may be related to the chemical composition, as both Temporary CB and Permanent Crown have a ceramic compound. As the tube voltage or gamma energy increases, the deviations decrease, eventually reaching a point where no excessive deviation is present at I-131 (364.5 keV). This behaviour is consistent with the expectation that the influence of the photoeffect on the attenuation properties decreases with increasing gamma energy and thus the elemental composition of the materials becomes less relevant.

Although all SLA printing is based on the same principle of curing resins under UV light, and our results suggest that resins without obvious additional compounds (e.g. glass or ceramic compounds) behave in a broadly similar manner, the specific compounds of commercially available resins can vary between manufacturers and are not always readily available. Therefore, we do not recommend extrapolating the results of this study to apparently similar materials without careful consideration.

## Conclusion

In summary, this study provides valuable insights into the behavior of various materials under radiometric measurements and offers a foundation for utilizing Formlabs 3D-printing materials in both nuclear medicine and X-ray imaging. However, researchers should be aware of the specific characteristics and deviations exhibited by certain materials, especially when working with dental materials or glass-compound materials at varying energy levels. These findings contribute to the advancement of accurate and reliable radiometric measurements and can facilitate the development of more precise radiological imaging and calibration techniques. Further research and refinement of experimental setups could lead to improved accuracy and broader applications in the field of radiometry.

## Declaration of Generative AI and AI-assisted technologies in the writing process

During the preparation of this work the author(s) used ChatGPT (OpenAI) in order to translate from german to english, as this work is based on a master thesis written in german. After using this tool/service, the author(s) reviewed and edited the content as needed and take(s) full responsibility for the content of the publication.

## Role of the funding source

While Formlabs kindly provided the majority of the used testobjects, it had no involvement in study design, data collection, analysis and interpretation of data, the writing of the report or in the decision to submit the data for publication.

## Declaration of Competing Interest

The authors declare that they have no known competing financial interests or personal relationships that could have appeared to influence the work reported in this paper.

## Figures and Tables

**Table A.7:** Mass attenuation coefficients [${\mathrm{cm}}^{2}/g$] in nuclear medicine for Tc-99 m (140.5 keV) and I-131 (364.5 keV). Uncertainties given in the parenthesis refer to the last digits. ☆ Values determined from direct comparative measurements.

	Tc-99 m	I-131
Material	140.5 keV	364.5 keV
BioMed Amber	0.1446(10)	0.0996(13)
BioMed Black	0.1457(11)	0.1021(10)
BioMed Clear	0.1441(13)	0.1016(12)
BioMed Elastic50A	0.1419(16)	0.0967(36)
BioMed Flexible80A	0.1400(22)	0.1035(49)
BioMed White hd	0.1412(12)	0.0981(47)
BioMed White ld	0.1443(25)	0.1077(36)
Black	0.1434(9)	0.0983(9)
Castable	0.1448(16)	0.0982(15)
Castable Wax	0.1435(13)	0.0972(18)
ClearV4	0.1456(12)	0.1004(8)
Custom Tray	0.1438(15)	0.1013(14)
Denture Base	0.1417(8)	0.0993(6)
Denture Teeth	0.1420(10)	0.0983(12)
Draft	0.1440(11)	0.1004(14)
Durable	0.1443(13)	0.0987(16)
Elastic50A	0.1444(20)	0.1010(15)
ESD	0.1443(11)	0.1029(25)
Flexible80A	0.1459(18)	0.0980(15)
Grey	0.1429(15)	0.0962(17)
Grey Pro	0.1452(13)	0.1015(8)
HighTemp	0.1370(23)	0.1007(10)
IBT	0.1438(11)	0.1009(23)
ModelV3	0.1438(23)	0.0966(11)
Nylon11	0.1473(11)	0.0996(28)
Nylon11CF	0.1428(11)	0.0996(12)
Nylon12	0.1489(19)	0.1056(14)
Nylon12GF	0.1437(15)	0.0961(20)
Permanent Crown	0.2065(16)	0.1000(12)
PMMA	0.1441(6)	0.1001(15)
PU650	0.1439(15)	0.0912(30)
PU1000	0.1464(11)	0.1060(27)
Rigid10k	0.1379(15)	0.0970(9)
Rigid4000	0.1419(10)	0.0975(9)
Temporary CB	0.2134(14)	0.0989(18)
Tough1500	0.1420(10)	0.1020(25)
Tough2000	0.1415(13)	0.1000(18)
TPU90A	0.1460(12)	0.1055(36)
White	0.1435(16)	0.1037(32)

**Table A.8:** Mass attenuation coefficients [${\mathrm{cm}}^{2}/g$] in X-ray imaging (tube voltages 60-110 kVp). Uncertainties given in the parenthesis refer to the last digits. ☆ Values determined from direct comparative measurements.

	Tube Voltage					
Material	60 kVp	70 kVp	80 kVp	90 kVp	99 kVp	110 kVp
BioMed Amber	0.2618(12)	0.2461(11)	0.2368(11)	0.2286(11)	0.2254(15)	0.2243(16)
BioMed Black	0.2668(20)	0.2518(16)	0.2423(15)	0.2347(15)	0.2343(31)	0.2310(20)
BioMed Clear	0.2529(12)	0.2408(12)	0.2324(13)	0.2247(12)	0.2224(17)	0.2201(15)
BioMed Elastic50A	0.2496(28)	0.2378(25)	0.2301(25)	0.2236(24)	0.2236(36)	0.2213(25)
BioMed Flexible80A	0.2538(19)	0.2398(31)	0.2335(14)	0.2259(14)	0.2259(28)	0.2237(19)
BioMed White hd	0.2967(29)	0.2789(35)	0.2644(31)	0.2551(29)	0.2593(81)	0.2469(56)
BioMed White ld	0.2603(12)	0.2451(15)	0.2356(20)	0.2280(18)	0.2260(24)	0.2244(21)
Black	0.2619(18)	0.2478(14)	0.2384(15)	0.2306(13)	0.2282(21)	0.2249(18)
Castable	0.2567(15)	0.2453(13)	0.2366(14)	0.2293(14)	0.2264(19)	0.2233(18)
Castable Wax	0.2575(14)	0.2469(15)	0.2374(13)	0.2294(13)	0.2252(19)	0.2229(17)
ClearV4	0.2581(17)	0.2441(18)	0.2355(16)	0.2251(17)	0.2275(29)	0.2243(21)
Custom Tray	0.2580(20)	0.2471(20)	0.2391(21)	0.2302(18)	0.2269(22)	0.2229(22)
Denture Base	0.2540(7)	0.2436(9)	0.2346(11)	0.2271(11)	0.2241(13)	0.2214(14)
Denture Teeth	0.2660(12)	0.2509(12)	0.2400(11)	0.2324(13)	0.2291(16)	0.2251(18)
Draft	0.2534(11)	0.2416(11)	0.2337(13)	0.2261(11)	0.2238(15)	0.2283(29)
Durable	0.2583(11)	0.2447(10)	0.2356(11)	0.2280(9)	0.2257(15)	0.2229(16)
Elastic50A	0.2573(42)	0.2433(32)	0.2345(31)	0.2264(30)	0.2246(32)	0.2214(28)
ESD	0.2570(11)	0.2437(9)	0.2351(10)	0.2271(12)	0.2242(15)	0.2218(14)
Flexible80A	0.2604(16)	0.2473(13)	0.2372(13)	0.2296(15)	0.2272(16)	0.2242(17)
Grey	0.2666(10)	0.2522(12)	0.2409(12)	0.2309(11)	0.2302(17)	0.2266(18)
Grey Pro	0.2688(17)	0.2531(14)	0.2425(13)	0.2339(14)	0.2313(18)	0.2282(16)
High Temp	0.2540(10)	0.2412(10)	0.2324(11)	0.2248(11)	0.2227(14)	0.2200(14)
IBT	0.2569(13)	0.2433(14)	0.2342(13)	0.2272(14)	0.2246(17)	0.2218(15)
ModelV3	0.2524(14)	0.2407(13)	0.2328(12)	0.2260(12)	0.2264(21)	0.2236(17)
Nylon11	0.2479(11)	0.2378(10)	0.2316(10)	0.2253(17)	0.2273(21)	0.2243(16)
Nylon11CF	0.2445(12)	0.2352(11)	0.2278(12)	0.2210(12)	0.2194(16)	0.2167(14)
Nylon12	0.2484(19)	0.2385(18)	0.2321(18)	0.2263(17)	0.2277(26)	0.2245(18)
Nylon12GF	0.4018(70)	0.3644(63)	0.3367(60)	0.3148(59)	0.3034(65)	0.2949(62)
Permanent Crown	1.349(19)☆	1.3034(79)☆	1.232(30)	1.160(39)	1.113(41)	1.056(45)
PMMA	0.2560(11)	0.2431(12)	0.2323(13)	0.2261(13)	0.2262(21)	0.2226(17)
PU650	0.2531(14)	0.2396(9)	0.2321(9)	0.2259(10)	0.2257(26)	0.2229(16)
PU1000	0.2517(13)	0.2406(13)	0.2324(12)	0.2258(12)	0.2261(28)	0.2233(18)
Rigid10k	0.452(15)	0.397(13)	0.363(11)	0.3373(97)	0.327(11)	0.3118(91)
Rigid4000	0.3410(48)	0.3096(42)	0.2903(37)	0.2730(40)	0.2667(45)	0.2600(43)
Temporary CB	1.477(25)☆	1.401(11)☆	1.3200(55)☆	1.223(44)	1.161(43)	1.103(56)
Tough1500	0.2533(15)	0.2420(17)	0.2336(13)	0.2267(14)	0.2274(23)	0.2242(19)
Tough2000	0.2840(23)	0.2667(21)	0.2559(24)	0.2460(19)	0.2449(28)	0.2401(24)
TPU90A	0.2562(16)	0.2439(15)	0.2360(15)	0.2288(14)	0.2295(24)	0.2257(18)
White	0.2628(19)	0.2489(15)	0.2406(15)	0.2329(14)	0.2326(23)	0.2292(19)

**Table B.9:** Linear attenuation coefficients [1/cm] in nuclear medicine for Tc-99 m (140.5 keV) and I-131 (364.5 keV). Uncertainties given in the parenthesis refer to the last digits. ☆ Values determined from direct comparative measurements.

	Tc-99 m	I-131
Material	140.5 keV	364.5 keV
BioMed Amber	0.1715(10)	0.1181(15)
BioMed Black	0.1730(11)	0.1212(12)
BioMed Clear	0.1685(14)	0.1188(14)
BioMed Elastic50A	0.1528(8)	0.1041(37)
BioMed Flexible80A	0.1537(23)	0.1137(54)
BioMed White hd	0.1739(13)	0.1208(58)
BioMed White ld	0.1694(28)	0.1264(41)
Black	0.1672(9)	0.1146(9)
Castable	0.1605(17)	0.1088(16)
Castable Wax	0.1704(14)	0.1154(21)
ClearV4	0.1703(10)	0.1174(7)
Custom Tray	0.1697(12)	0.1195(14)
Denture Base	0.1682(9)	0.1179(7)
Denture Teeth	0.1676(11)	0.1160(14)
Draft	0.1700(12)	0.1186(16)
Durable	0.1624(14)	0.1111(18)
Elastic50A	0.1565(12)	0.1095(10)
ESD	0.1633(12)	0.1164(28)
Flexible80A	0.1616(19)	0.1086(16)
Grey	0.1669(17)	0.1123(19)
Grey Pro	0.1692(14)	0.1182(8)
HighTemp	0.1639(27)	0.1205(11)
IBT	0.1621(11)	0.1138(25)
ModelV3	0.1711(27)	0.1149(13)
Nylon11	0.1482(10)	0.1002(28)
Nylon11CF	0.1497(10)	0.1045(12)
Nylon12	0.1452(16)	0.1030(12)
Nylon12GF	0.1809(16)	0.1210(24)
Permanent Crown	0.3046(20)	0.1475(17)
PMMA	0.1705(6)	0.1184(17)
PU650	0.1649(16)	0.1045(34)
PU1000	0.1675(11)	0.1213(31)
Rigid10k	0.2341(26)	0.1646(15)
Rigid4000	0.1891(12)	0.1299(12)
Temporary CB	0.3180(19)	0.1474(26)
Tough1500	0.1628(11)	0.1170(28)
Tough2000	0.1679(15)	0.1187(21)
TPU90A	0.1648(11)	0.1191(41)
White	0.1672(18)	0.1208(36)

**Table B.10:** Linear attenuation coefficients [1/cm] in X-ray imaging (tube voltages 60-110 kVp). Uncertainties given in the parenthesis refer to the last digits. ☆ Values determined from direct comparative measurements.

	Tube Voltage					
Material	60 kVp	70 kVp	80 kVp	90 kVp	99 kVp	110 kVp
BioMedAmber	0.3106(11)	0.2920(9)	0.2809(10)	0.2712(10)	0.2674(16)	0.2661(17)
BioMed Black	0.3167(22)	0.2989(16)	0.2877(15)	0.2786(16)	0.2782(36)	0.2743(22)
BioMed Clear	0.2957(9)	0.2816(10)	0.2718(12)	0.2628(11)	0.2601(17)	0.2574(16)
BioMed Elastic50A	0.2688(15)	0.2561(11)	0.2478(13)	0.2408(12)	0.2408(30)	0.2384(19)
BioMed Flexible80A	0.2787(18)	0.2633(33)	0.2564(12)	0.2481(12)	0.2481(29)	0.2457(20)
BioMed White hd	0.3655(32)	0.3436(39)	0.3257(35)	0.3143(33)	0.3194(99)	0.3041(67)
BioMed White ld	0.3056(2)	0.2877(12)	0.3766(20)	0.2676(17)	0.2653(26)	0.2634(21)
Black	0.3054(17)	0.2890(12)	0.2780(14)	0.2689(12)	0.2661(23)	0.2622(19)
Castable	0.2845(10)	0.2719(8)	0.2622(10)	0.2541(12)	0.2509(18)	0.2475(18)
Castable Wax	0.3058(10)	0.2932(12)	0.2820(9)	0.2724(10)	0.2686(18)	0.2647(17)
ClearV4	0.3019(7)	0.2855(11)	0.2754(8)	0.2633(11)	0.2661(30)	0.2623(18)
Custom Tray	0.3044(9)	0.2916(10)	0.2821(14)	0.2717(10)	0.2677(18)	0.2630(18)
Denture Base	0.3015(5)	0.2891(9)	0.2785(12)	0.2695(11)	0.2660(15)	0.2628(16)
Denture Teeth	0.3141(13)	0.2962(13)	0.2833(11)	0.2744(14)	0.2705(18)	0.2657(20)
Draft	0.2992(6)	0.2853(8)	0.2759(12)	0.2669(9)	0.2642(14)	0.2696(33)
Durable	0.2907(11)	0.2754(9)	0.2652(11)	0.2566(9)	0.2540(16)	0.2509(17)
Elastic50A	0.2789(30)	0.2637(12)	0.2542(14)	0.2454(12)	0.2434(19)	0.2400(18)
ESD	0.2908(11)	0.2757(9)	0.2660(9)	0.2560(11)	0.2536(16)	0.2509(15)
Flexible80A	0.2885(13)	0.2740(9)	0.2628(9)	0.2543(12)	0.2517(15)	0.2484(17)
Grey	0.3113(8)	0.2945(12)	0.2813(12)	0.2696(10)	0.2686(19)	0.2646(19)
Grey Pro	0.3132(18)	0.2948(13)	0.2825(13)	0.2725(15)	0.2694(19)	0.2659(18)
High Temp	0.3040(7)	0.2886(8)	0.2781(9)	0.2690(9)	0.2665(15)	0.2633(14)
IBT	0.2897(8)	0.2743(11)	0.2641(10)	0.2562(11)	0.2533(16)	0.2501(14)
ModelV3	0.3002(16)	0.2863(14)	0.2769(13)	0.2689(13)	0.2693(25)	0.2660(19)
Nylon11	0.2495(9)	0.2393(8)	0.2331(7)	0.2267(16)	0.2287(21)	0.2257(15)
Nylon11CF	0.2564(7)	0.2467(5)	0.2389(8)	0.2318(9)	0.2301(14)	0.2273(12)
Nylon12	0.2423(7)	0.2326(6)	0.2264(7)	0.2207(6)	0.2221(20)	0.2189(14)
Nylon12GF	0.5059(83)	0.4588(74)	0.4239(71)	0.3963(70)	0.3820(78)	0.3713(74)
Permanent Crown	2.011(29)☆	1.942(11)☆	1.817(43)	1.711(57)	1.642(61)	1.558(65)
PMMA	0.3020(11)	0.2876(12)	0.2748(13)	0.2675(13)	0.2676(24)	0.2633(18)
PU650	0.2899(14)	0.2745(8)	0.2659(9)	0.2588(10)	0.2586(29)	0.2553(17)
PU1000	0.2880(10)	0.2753(11)	0.2659(10)	0.2583(11)	0.2587(31)	0.2554(18)
Rigid10k	0.768(25)	0.674(22)	0.617(19)	0.573(17)	0.555(19)	0.529(16)
Rigid4000	0.4544(63)	0.4125(55)	0.3868(48)	0.3638(53)	0.3553(60)	0.3464(57)
Temporary CB	2.201(37)☆	2.088(15)☆	1.9672(72)☆	1.822(65)	1.730(64)	1.644(82)
Tough1500	0.2904(14)	0.2774(17)	0.2678(12)	0.2599(13)	0.2607(25)	0.2571(20)
Tough2000	0.3371(26)	0.3165(24)	0.3037(28)	0.2920(21)	0.2906(32)	0.2849(28)
TPU90A	0.2891(13)	0.2752(12)	0.2663(13)	0.2582(11)	0.2590(25)	0.2547(18)
White	0.3062(20)	0.2901(14)	0.2804(14)	0.2714(13)	0.2711(25)	0.2671(21)

## Data Availability

The code used to extract the data is distributed by the authors as open-source. The patient data can be made available on request due to privacy/ethical restrictions.
